# Fibronectin Binding Is Required for Acquisition of Mesenchymal/Endothelial Differentiation Potential in Human Circulating Monocytes

**DOI:** 10.1155/2012/820827

**Published:** 2012-11-01

**Authors:** Noriyuki Seta, Yuka Okazaki, Keisuke Izumi, Hiroshi Miyazaki, Takashi Kato, Masataka Kuwana

**Affiliations:** ^1^Division of Rheumatology, Department of Internal Medicine, Keio University School of Medicine, 35 Shinanomachi, Shinjuku-ku, Tokyo 160-8582, Japan; ^2^Innovative Drug Research Laboratories, Research Division, Kyowa Hakko Kirin Co., Ltd., 3 Miyahara, Takasaki, Gunma 370-1295, Japan; ^3^Department of Biology, School of Education, Waseda University, 2-2 Wakamatsu, Shinjuku-ku, Tokyo 162-8480, Japan

## Abstract

We previously reported monocyte-derived multipotential cells (MOMCs), which include progenitors capable of differentiating into a variety of mesenchymal cells and endothelial cells. *In vitro* generation of MOMCs from circulating CD14^+^ monocytes requires their binding to extracellular matrix (ECM) protein and exposure to soluble factor(s) derived from circulating CD14^−^ cells. Here, we investigated the molecular factors involved in MOMC generation by examining the binding of monocytes to ECM proteins. We found that MOMCs were obtained on the fibronectin, but not on type I collagen, laminin, or poly-L-lysine. MOMC generation was followed by changes in the expression profiles of transcription factors and was completely inhibited by either anti-**α**_5_ integrin antibody or a synthetic peptide that competed with the RGD domain for the **β**_1_-integrin binding site. These results indicate that acquisition of the multidifferentiation potential by circulating monocytes depends on their binding to the RGD domain of fibronectin via cell-surface **α**_5_ **β**_1_ integrin.

## 1. Introduction

Circulating CD14^+^ monocytes originate from hematopoietic stem cells in the bone marrow and are heterogeneous in terms of their surface markers, phagocytic capacity, and differentiation potentials [1]. Until recently, it was believed that the differentiation potential of monocytes was restricted to cells that function as phagocytes and/or antigen-presenting cells, including macrophages, dendritic cells, and osteoclasts, but recent accumulating evidence indicates that circulating monocytes have the potential to differentiate into a variety of cell types other than phagocytes [2–6]. We recently identified a peripheral blood-derived cell population that has a fibroblast-like morphology in culture and a unique phenotype positive for CD14, CD45, CD34, and type I collagen (collagen (I)) [7]. These cells originate from circulating CD14^+^ monocytes and include primitive cells that can differentiate into cells with morphologic, phenotypic, and functional features of several distinct mesenchymal cell types, including bone, cartilage, fat, and skeletal and cardiac muscle, as well as neurons and endothelium; thus, they are termed monocyte-derived multipotential cells (MOMCs) [7–10]. It is thus plausible that circulating monocytes are involved in tissue remodelling and regeneration through MOMC differentiation under physiologic and pathogenic conditions *in vivo*. 

MOMCs are obtained in 7-day cultures of peripheral blood mononuclear cells (PBMCs) on fibronectin-coated plastic plates with 10% fetal bovine serum (FBS) as the only source of growth factors. A series of our experiments indicated that the *in vitro* generation of MOMCs from circulating CD14^+^ monocytic progenitors requires their binding to extracellular matrix (ECM) components such as fibronectin and exposure to soluble factor(s) derived from peripheral blood CD14^−^ cells [7], although the detailed mechanisms involved in this process remain unknown. In this study, we examined the molecular factors required for the *in vitro* generation of MOMCs by focusing on their binding to ECM proteins.

## 2. Materials and Methods

### 2.1. Cultures for MOMC Generation

PBMCs obtained from 10 Japanese healthy volunteers aged 22–38 were used for MOMC generation cultures as described previously [7]. Briefly, PBMCs (2 × 10^6^ cells/mL) were cultured in low-glucose Dulbecco's modified Eagle's medium (DMEM) supplemented with 10% FBS (JRH Bioscience, Lenexa, KS) for 7 days on plastic plates coated with fibronectin without additional growth factors. We used two different batches of commercially available fibronectin preparation isolated from pooled human plasma: one purchased from Sigma, St. Louis, MO, USA, (catalog number F-0895, lot number 71K7617) and the other from BD Biosciences, San Diego, CA, USA (catalog number 354249, lot number 007591). MOMC generation cultures were also performed on plastic plates coated with collagen (I), laminin, or poly-L-lysine (BD Biosciences). The medium containing floating cells was exchanged with fresh medium every 3 days. The number and morphology of the adherent cells were assessed under an inverted microscope (IX81: Olympus, Tokyo, Japan). To evaluate the proliferative capacity of the adherent cells, the cells recovered by incubation with 0.25% trypsin were replated on new plastic plates coated with collagen (I) or fibronectin and maintained under the same culture conditions for up to 6 passages. All blood samples were obtained after the subjects gave their written informed consent, as approved by the Institutional Review Board.

In some experiments, CD14^+^ monocytes and CD14^−^ PBMCs were separated from PBMCs using MACS column separation (Miltenyi Biotech, Bergisch Gladbach, Germany). The conditioned medium was prepared by culturing CD14^−^ PBMCs on fibronectin overnight as described previously [7]. The CD14^+^ monocytes (2 × 10^5^ cells/mL) were cultured with CD14^−^ PBMC-conditioned medium on fibronectin-or collagen (I)-coated plastic plates for 7 days. Anti-*α*_3_ integrin, anti-*α*_4_ integrin, or anti-*α*_5_ integrin monoclonal antibody (mAb) (Beckman Coulter, Fullerton, CA, USA) was added at a final concentration of 20 *μ*g/mL at the initiation of culture and added again every other day. The same culture was also conducted in the presence of an RGD domain peptide (GRGDSP) or its corresponding control peptide (GRGESP; Takara Bio, Shiga, Japan) or a connecting segment (CS)-1 domain peptide (EILDV) or its corresponding control peptide (EILAV; Invitrogen, Carlsbad, CA, USA) at a final concentration of 500 *μ*g/mL. The results were expressed as a proportion (%) of the number of adherent cells in a given culture to the number of the adherent cells in the control culture without any peptide or vehicle.

### 2.2. Flow Cytometric Analysis

Cells were stained with FITC-conjugated anti-CD34 mAb (Beckman Coulter) or PC-5-conjugated anti-CD14 mAb (BD Biosciences). For staining of collagen (I), the cells were permeabilized and fixed using IntraPrep permeabilization reagent (Beckman Coulter) and then incubated with unconjugated anti-collagen (I) (Millipore, Billerica, MA, USA) followed by staining with FITC-conjugated goat anti-mouse IgG F(ab′)_2_ (Beckman Coulter). Stained cells were analyzed on a FACS Calibur flow cytometer using CellQuest software (BD Biosciences). Viable cells were identified by gating based on forward and side scatters, and the data were shown as histograms. 

### 2.3. Analysis of mRNA Expression

The expression of mRNA was examined using reverse transcription (RT) and semiquantitative polymerase-chain reaction (PCR) as described previously [7]. Briefly, first-strand cDNA was synthesized from 1 *μ*g of total RNA, and aliquots of the cDNA (50 ng of total RNA equivalent) were subjected to PCR. Primer sequences and optimal PCR conditions were described previously [7, 10] or shown in Table 1. Pooled PBMCs, umbilical vein endothelial cells, skin fibroblasts, the promyelocytic leukemia cell line HL-60, and the acute monocytic leukemia cell line THP-1 were used as positive controls for specific amplifications. The PCR products were resolved by electrophoresis on 2% agarose gels and visualized by ethidium bromide staining. The intensity of individual bands was semiquantitatively analyzed using the Image/J software [11]. The relative expression level of individual genes was normalized to the expression of GAPDH.

The mRNA expression of selected genes was further evaluated using a quantitative TaqMan real-time PCR system (Applied Biosystems, Foster City, CA, USA). All primers and probes were purchased from Applied Biosystems. The gene expression was standardized based on serial amounts of cDNA prepared from fibroblasts for early growth response-1 (Egr-1) and HL-60 cells for acute myelogenous leukemia-1 (AML-1). The relative expression levels of individual genes were normalized to the expression level of GAPDH.

### 2.4. *In Vitro* Differentiation into Mesenchymal and Endothelial Lineages

The adherent cells obtained in a series of MOMC generation cultures were replated on fibronectin- or collagen (I)-coated chamber slides (BD Biosciences) in high-glucose DMEM with 10% FBS and grown to semiconfluence. The cells were then cultured under conditions known to induce the differentiation of MOMCs into mesenchymal and endothelial lineages [7, 10]. Their differentiation into osteoblasts, chondroblasts, and adipocytes was evaluated by alizarin red staining, immunostaining for type II collagen (collagen (II)), and oil red O staining, respectively [7]. The expression of lineage-specific transcription factors was evaluated by fluorescent double staining of Cbfa1 and CD45 for osteoblasts and Sox-9 and CD45 for chondroblasts [7]. Differentiation into the endothelial lineage was evaluated by the fluorescent staining of endothelial nitric oxide synthase (eNOS) or Tie-2 in combination with 4′,6-diamidino-2-phenylindole, and dihydrochloride [10]. 

### 2.5. Statistical Analysis

Comparisons between two groups were tested for statistical significance using the nonparametric Mann-Whitney *U* test or paired *t*-test where applicable.

## 3. Results and Discussion

### 3.1. MOMC Generation Requires Binding to Fibronectin

**Figure 1:**
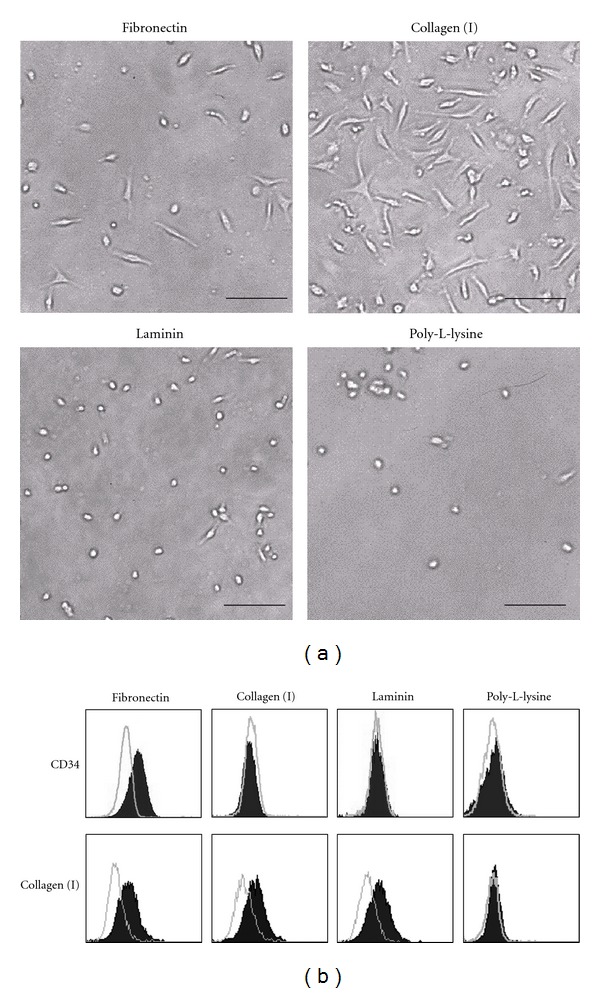
MOMC generation cultures on fibronectin, collagen (I), and laminin, or poly-L-lysine. (a) PBMCs (2 × 10^6^) were cultured for 7 days on 24-well plastic plates coated with fibronectin (Sigma), collagen (I), laminin, or poly-L-lysine. The medium containing floating cells was exchanged with fresh medium every 3 days. The adherent cells were observed with an inverted microscope. Bars: 100 *μ*m. (b) Adherent cells recovered were subjected to flow cytometry for evaluation of cell surface expression of CD34 and intracellular expression of collagen (I). The expression of CD34 and collagen (I) is shown as shaded histograms, while open histograms represent staining with isotype-matched control mAb. The results shown are representative of 5 independent experiments.

To evaluate whether fibronectin is essential for the MOMC generation, PBMCs from five independent donors were cultured on a variety of ECM proteins, including fibronectin, collagen (I), and laminin. Poly-L-lysine was used as a control for cell adherence. Concordant findings were obtained from all donors and 7-day cultures on fibronectin (Sigma), collagen (I), or laminin resulted in the appearance of spindle-shaped cells (Figure 1(a)). In contrast, only a few round cells were observed in the culture on poly-L-lysine. The collagen (I) cultures yielded 4.1 ± 0.8-fold more adherent cells than did the fibronectin cultures (*P* < 0.05). Flow cytometric analysis of the adherent CD14^+^ cells revealed that the cells cultured on fibronectin coexpressed CD34, but those cultured on collagen (I), laminin, or poly-L-lysine did not, whereas intracellular collagen (I) was detected in the cells cultured on any type of ECM protein (Figure 1(b)). These findings indicate that adherent cells expressing CD34 and collagen (I), the typical features of MOMCs [6], were obtained exclusively in the PBMCs cultured on fibronectin. Consistent results were obtained from experiments using fibronectin purchased from Sigma and BD Biosciences. In addition, effect of FBS-derived fibronectin contained in the culture medium was negligible in our MOMC generation cultures.

**Figure 2:**
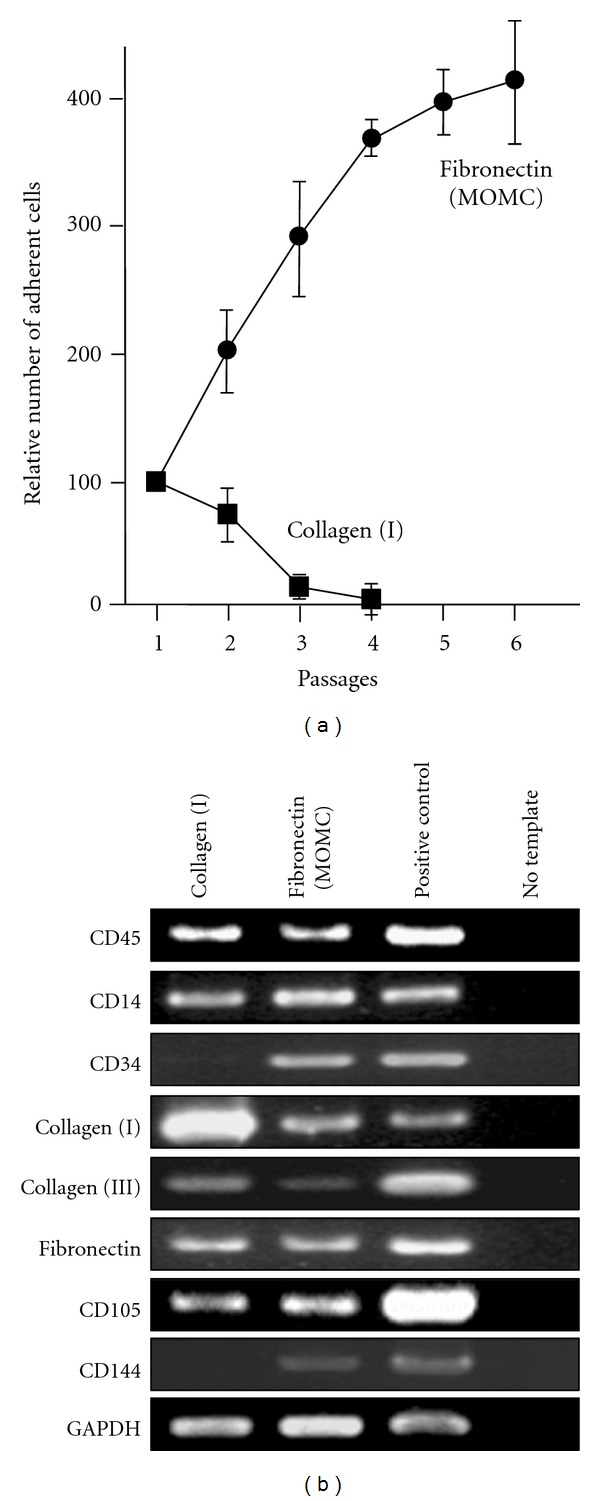
Proliferative capacity and gene expression profiles of adherent cells obtained in the MOMC generation cultures on fibronectin or collagen (I). (a) PBMCs (2 × 10^6^) were cultured on 24-well plastic plates coated with fibronectin (Sigma) or collagen (I). The medium containing floating cells was exchanged with fresh medium every 3 days. At day 7, all adherent cells were detached, counted, and replated on plates coated with fibronectin (Sigma) or collagen (I). Results are expressed as proportion of the number of adherent cells recovered at each passage to the number of adherent cells recovered at the first passage. The mean and standard deviation of 5 independent experiments are shown. (b) Adherent cells recovered were subjected to reverse transcription and semi-quantitative PCR for evaluation of mRNA expression. Positive controls for the specific amplification included pooled PBMCs for CD14, CD45, and GAPDH; cultured human umbilical vein endothelial cells for CD34, CD105, and CD144; cultured skin fibroblasts for collagen (I), collagen (III), and fibronectin. The results are representative of 4 independent experiments.

To further examine the characteristics of the CD14^+^CD34^−^collagen (I)^+^ cells obtained in the PBMC cultures on collagen (I), their proliferative capacity, gene expression profile, and differentiation potential were compared with those of the CD14^+^CD34^+^ collagen (I)^+^ MOMCs obtained in the culture on fibronectin (Sigma). MOMCs maintained their proliferative capacity on fibronectin-coated plates for up to 6 passages, whereas the cells obtained on collagen (I) were unable to proliferate after being passaged onto collagen (I)-coated plates and died after the second passage (Figure 2(a)). The failure of cells obtained in the collagen (I) culture to expand was also observed after their passage onto plates coated with fibronectin (Sigma and BD Biosciences). It has been generally believed that monocytes lack proliferative capacity, but recent reports have shown that subpopulations of circulating monocytes are capable of proliferating in certain *in vitro *culture systems [12] or pathologic conditions [13]. In this regard, we have reported that exposure of circulating monocytes to fibronectin resulted in acquisition of proliferative capacity using flow cytometric analysis of CFSE-labeled monocytes and immunohistochemical detection of BrdU incorporation in cultured monocytes [7]. It is likely that collagen (I) lacks this potential. 

Representative semi-quantitative PCR results of the adherent cells obtained in the collagen (I) or fibronectin cultures are shown in Figure 2(b). Cells obtained in the collagen (I) cultures lacked CD34 and CD144 expression and showed increased expression levels of collagens including collagen (I) and type III collagen (collagen (III)), compared with the MOMCs obtained in the fibronectin cultures. Semi-quantitative analysis for mRNA expression in five independent samples revealed that difference in the expression levels of collagen (I) and (III) between cultures on collagen (I) and fibronectin (Sigma) was statistically significant (15.6 ± 1.8 versus 3.8 ± 0.5 and 2.1 ± 0.8 versus 0.6 ± 0.3; *P* < 0.05 for both comparisons). Consistent results were obtained in experiments using samples from four additional independent donors. Additional experiments using fibronectin obtained from BD Biosciences confirmed difference in expression profiles between collagen (I)-or fibronectin-coated cultures. 

Finally, cells obtained in the collagen (I) cultures were applied to lineage-specific induction cultures towards osteoblasts, chondrocytes, adipocytes, and endothelial cells either on fibronectin- or collagen (I)-coated slides, but nearly all the cells were detached during the culture. The small number of cells adhering to the plates on day 7 was subjected to fluorescent staining for lineage-specific markers: Cbfa1 for osteogenesis, PPAR*γ* for adipogenesis, and eNOS or Tie-2 for endothelial differentiation, but no specific staining was detected. In contrast, MOMCs simultaneously prepared from the same donors with fibronectin (either from Sigma or BD Biosciences) were able to differentiate into individual cell lineages (data not shown). 

These results together indicate that the CD14^+^CD34^−^ collagen (I)^+^ cells obtained in the collagen (I) cultures were distinct from MOMCs, based on their lack of proliferative capacity and inability to differentiate into mesenchymal/endothelial lineages. In this regard, Jacob and coworkers reported that treating monocytes with fibronectin and collagen (I) results in different expression levels of monocytic markers, matrix metalloproteinases, and endocytosis capacity [14]. Taken together, the MOMC generation in PBMC cultures requires the specific binding of monocytes to fibronectin.

Our findings indicate that the microenvironment provided by ECM proteins can modulate monocyte differentiation. Since the interstitial structures around blood vessels are rich in fibronectin [15], monocytes infiltrating injured tissues would encounter fibronectin, and might contribute to tissue regeneration through their differentiation into MOMCs. In contrast, monocytes that are exposed to the collagen (I)-enriched dense connective tissue might produce large amounts of collagens to repair the damaged tissue. Therefore, it is likely that the different distributions of ECM proteins in tissues control the differentiation capacity of the circulating monocytic progenitors. These heterogeneous responses of monocytes could be explained by the different signal pathways activated by ECM proteins [16]. Alternatively, the cellular origin of the circulating monocytes attaching to fibronectin or collagen (I) might be different. 

### 3.2. Binding of CD14^+^ Monocytes to Fibronectin versus Collagen (I) Results in the Upregulation of Different Sets of Transcription Factors

**Figure 3:**
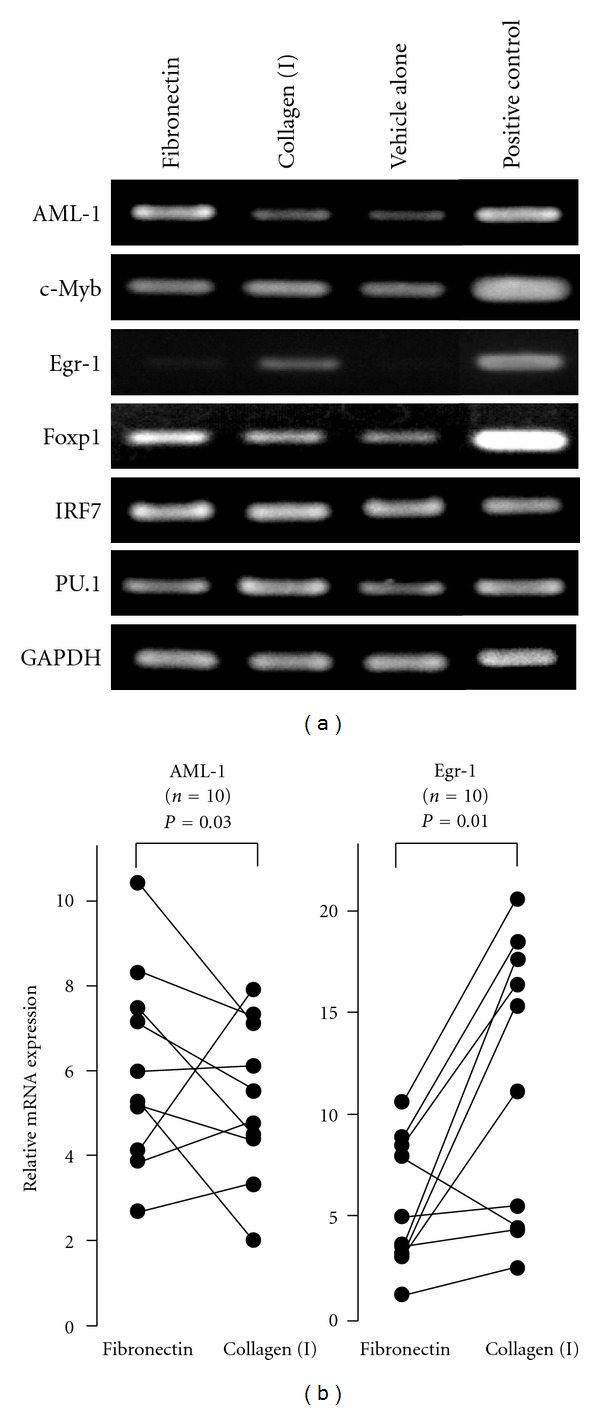
Expression profiles of transcription factors in monocytes exposed to fibronectin or collagen (I) for 24 hours. (a) MACS-sorted CD14^+^ monocytes (4 × 10^5^) were cultured with CD14^−^ PBMC-conditioned medium on 24-well plastic plates coated with fibronectin (Sigma), collagen (I), or vehicle alone for 24 hours. Then, adherent cells were recovered and subjected to reverse transcription and semi-quantitative PCR for evaluation of mRNA expression. Positive controls for the specific amplification included cultured skin fibroblasts for Egr-1, HL-60 for PU.1, c-Myb, and AML-1, and THP-1 for IRF-7 and Foxp1. The results shown are representative of 5 independent experiments. (b) Quantitative Taqman real-time PCR examining gene expression levels of Egr-1 and AML-1 in adherent cells obtained from cultures of CD14^+^ monocytes with CD14^−^ PBMC-conditioned medium for 24 hours on fibronectin (BD Biosciences) or collagen (I). Results obtained from 10 independent healthy donors are shown. Differences in expression levels are tested by paired *t*-test.

When circulating monocytes are recruited to tissues across endothelial and ECM barriers, integrins mediate their adhesion to the ECM proteins as well as interactions that control complex cellular functions, such as proliferation, differentiation, and survival, by regulating gene expression [17]. To evaluate the mechanisms responsible for the differentiation of monocytes into different cell types upon their exposure to fibronectin and collagen (I), we focused on the gene expression of transcription factors involved in the cellular functions and differentiation of monocytes [18]. The expression profiles of the transcription factors AML-1, c-Myb, Egr-1, Foxp1, interferon regulatory factor-7 (IRF-7), and PU.1 were examined by semi-quantitative PCR in CD14^+^ monocytes cultured in CD14^−^ PBMC-conditioned medium on plastic plates coated with fibronectin (Sigma), collagen (I), or vehicle alone for 24 hours. Upon exposure to fibronectin, the cells showed up-regulated AML-1 expression, and upon exposure to collagen (I), they showed up-regulated Egr-1 expression (Figure 3(a)). Semi-quantitative analysis for mRNA expression in 5 independent samples revealed that difference in the expression levels of AML-1 and Egr-1 between cultures on fibronectin and collagen (I) was statistically significant (2.5 ± 0.7 versus 0.6 ± 0.4 and 0.10 ± 0.09 versus 0.56 ± 0.35; *P* < 0.05 for both comparisons). Moreover, real-time PCR was carried out to confirm these findings using 10 independent samples and another batch of fibronectin (BD Biosciences). As shown in Figure 3(b), AML-1 expression was increased, and Egr-1 expression was decreased in cultures with fibronectin than in those with collagen (I) (*P* = 0.03 and 0.01, resp.). 

AML-1, also termed Runx1, is widely expressed in hematopoietic lineages, and acts as either a transcriptional repressor or an activator on a variety of hematopoietic genes [19]. It has been shown that AML-1 is directly involved in the differentiation and maturation of endothelial progenitor cells [20], which have characteristics similar to MOMCs in terms of CD34 expression and the potential to differentiate into endothelial cells and cells in mesenchymal lineages [21]. On the other hand, Egr-1 is a master regulator controlling the expression of a wide variety of genes implicated in cell growth, differentiation, and survival. A recent study indicated that Egr-1 is involved in wound healing and tissue fibrosis by regulating transforming growth factor-*β*-dependent physiological and pathological matrix remodeling [22], although the cellular functions associated with Egr-1 in monocytes remain uncertain. Further studies are necessary to investigate the roles of these up-regulated transcription factors in the circulating monocytes' acquisition of new cellular functions and differentiation potentials. 

### 3.3. An Interaction between the RGD Domain of Fibronectin and *α*_5_ *β*_1_ Integrin Triggers MOMC Generation

**Figure 4:**
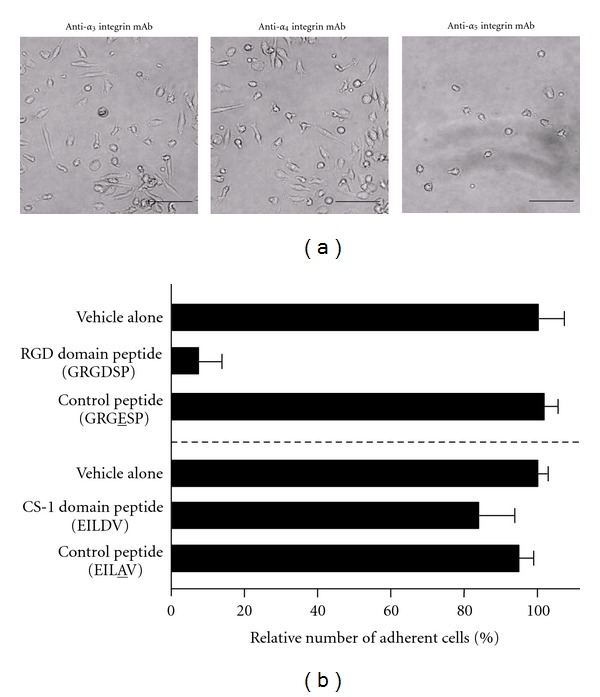
Effects of anti-*α* integrin mAb, RGD domain peptide, or CS-1 domain peptide on MOMC generation. (a) MACS-sorted CD14^+^ monocytes (4 × 10^5^) were cultured with CD14^−^ PBMC-conditioned medium on 24-well plastic plates coated with fibronectin (Sigma) for 7 days. Anti-*α*_3_ integrin, anti-*α*_4_ integrin, or anti-*α*_5_ integrin mAb was added at the initiation of cultures and added again every other day. The medium containing floating cells was exchanged with fresh medium at day 3. Adherent cells were observed with an inverted microscope. Bars: 100 *μ*m. (b) An RGD domain peptide (GRGDSP) or its corresponding control peptide (GRGESP) or a CS-1 domain peptide (EILDV) or its corresponding control peptide (EILAV) was added at the initiation of cultures and added again every other day. The number of adherent cells obtained at day 7 was counted. The results are expressed as proportion of the number of adherent cells in individual cultures to the number of adherent cells in the control culture without peptide or vehicle. The results shown are the mean and standard deviation of 3 independent experiments.

Since fibronectin is a ligand for various *β*_1_ integrins, including *α*_3_ *β*_1_, *α*_4_ *β*_1_, and *α*_5_ *β*_1_, we next examined which *β*_1_ integrin is involved in the interaction between monocytes and fibronectin during MOMC generation. Flow cytometric analysis using PBMCs from 10 healthy donors revealed that nearly all the circulating CD14^+^ monocytes expressed the *α*_4_ *β*_1_ and *α*_5_ *β*_1_ integrins (>95%), but not *α*_3_ *β*_1_ integrin (data not shown). We then assessed the effect of blocking these interactions using anti-*α*_3_ integrin, anti-*α*_4_ integrin, or anti-*α*_5_ integrin mAbs, on MOMC generation cultures consisting of CD14^+^ monocytes with CD14^−^ cell-conditioned medium on fibronectin (Sigma or BD Biosciences). In this experiment, the anti-*α*_3_ integrin mAb served as a control. Treatment with the anti-*α*_5_ integrin mAb completely inhibited the generation of spindle-shaped cells, resulting in the recovery of a very small number of adherent round cells (Figure 4(a)). In contrast, the anti-*α*_4_ integrin mAb had no effect on the appearance of the spindle-shaped cells. As a result, the number of adherent cells in the culture with anti-*α*_5_ integrin mAb was significantly less than the number in the culture with anti-*α*_4_ integrin mAb (16.6 ± 0.55 versus 112.7 ± 0.42; *P* < 0.05). The concordant results were obtained from cultures on either batch of fibronectin. Adherent cells obtained from the culture with anti-*α*_4_ integrin mAb were confirmed to be positive for CD34 and collagen (I) and to have mesenchymal/endothelial differentiation potentials. Consistent results were obtained in experiments using samples from five independent donors. These results indicate that an interaction between *α*_5_ *β*_1_ integrin and fibronectin is essential for the MOMC generation. 

Fibronectin is known to bind *β*_1_ integrin through two distinct functional domains, the RGD and CS-1 domains [23]. Therefore, we next examined the effects of peptides representing the RGD or CS-1 fibronectin domain, which would compete for fibronectin binding to integrin in MOMC generation cultures (Figure 4(b)). Corresponding mutated peptides that lacked the ability to bind integrin were used as controls. As a result, the appearance of spindle-shaped cells was suppressed only in the presence of the RGD domain peptide. The difference in the adherent cells in cultures with the RGD domain peptide and the control peptide was statistically significant (*P* < 0.05). Moreover, the adherent cells obtained in the presence of the CS-1 domain or control peptide had the typical features of MOMCs: CD34^+^collagen (I)^+^ and the potential to differentiate into mesenchymal/endothelial lineages. These results were reproducible in samples from five independent donors. Therefore, the acquisition of the mesenchymal/endothelial differentiation potential in circulating monocytes depends on their binding to the RGD domain of fibronectin via *α*_5_ *β*_1_ integrin on their cell surface. In the case of tissue injury, this process might be enhanced by infiltrating inflammatory cells that produce CC chemokines, such as monocyte inflammatory protein-1*α*, RANTES, and monocyte chemoattractant protein-1, which have been shown to upregulate the avidity of *α*_5_ *β*_1_ integrin on the surface of monocytes [24]. However, the detailed processes of intracellular signal transduction via *α*_5_ *β*_1_ integrin remain unclear. 

One of the limitations of this study is use of commercially available fibronectin preparations purified from human plasma, which were likely to contain degradation products. It has been reported that fibronectin fragments produced by proteolytic digestion are more powerful inducers for secretion of cytokines by monocytes in comparison with intact fibronectin, indicating that biological activities of fibronectin are dependent on its degradation status [25, 26]. Therefore, it is possible that the role of fibronectin in MOMC generation is mediated through fibronectin fragments with capacity to bind to *α*_5_ *β*_1_ integrin, rather than intact fibronectin. Further studies using intact and fragmented fibronectin preparations purified by affinity chromatography are necessary to clarify the roles of individual fibronectin fragments on differentiation potentials of circulating monocytes. 

## 4. Conclusion

Circulating monocytes are recruited to tissue and differentiate into cells with various functional properties in response to organ-specific cues provided by the surrounding environment. Our present findings indicate that acquisition of the mesenchymal/endothelial differentiation potential by circulating CD14^+^ monocytes depends on their binding to fibronectin via *α*_5_ *β*_1_ integrin. This external signal would induce specific intracellular signals in the infiltrating monocytes, resulting in modulation of the expression profiles of transcription factors, which induce the monocytes' differentiation into MOMCs. Further studies examining the intracellular signaling induced by the fibronectin-*α*_5_ *β*_1_ integrin interaction during MOMC generation will increase our understanding about how circulating CD14^+^ monocytic progenitors acquire mesenchymal/endothelial differentiation potentials.

## Figures and Tables

**Table 1:** Primer sequences and PCR conditions.

Gene		Primer sequences (5′ to 3′)	Annealing temperature (°C)	PCR cycles
AML-1	Sense	ACAAGGCAGATCCAACCATC	63	33
	Antisense	GTCGGGGAGTAGGTGAAGG		

c-Myb	Sense	CTGCTCACACCACTGGGAAG	63	38
	Antisense	CGAGCTTGACTGGAAGATGTC		

Egr-1	Sense	TGACCGCAGAGTCTTTTCCT	63	35
	Antisense	GATGAGCTGGGACTGGTAGC		

Foxp1	Sense	ACGCCTACTGCACACCTCTC	63	30
	Antisense	CTTCAGCTTCCTCTGGATCG		

IRF-7	Sense	ACTGTGACACCCCCATCTTC	63	30
	Antisense	GCTCCATAAGGAAGCACTCG		

PU.1	Sense	AGATGCACGTCCTCGATACC	63	30
	Antisense	GCTTGGACGAGAACTGGAAG		

AML-1: acute myelogenous leukemia-1, Egr-1: early growth response-1, IRF-7: interferon regulatory factor-7.
